# Trajectories of sickness absence and disability pension before and after colorectal cancer: A Swedish longitudinal population-based matched cohort study

**DOI:** 10.1371/journal.pone.0245246

**Published:** 2021-01-07

**Authors:** Lingjing Chen, Kristina A. E. Alexanderson

**Affiliations:** Division of Insurance Medicine, Department of Clinical Neuroscience, Karolinska Institutet, Stockholm, Sweden; University of Oxford, UNITED KINGDOM

## Abstract

**Objectives:**

Working-aged colorectal cancer (CRC) patients have a much better survival, indicating the importance of their future work situation. We investigated trajectories of sickness absence and disability pension (SADP) days before and after CRC diagnosis, and risk factors associated with different trajectories.

**Methods:**

A longitudinal, population-based matched cohort study of 4735 CRC survivors in Sweden aged 19–62 when first diagnosed with CRC in 2008–2011, and 18,230 matched references was conducted, using microdata linked from several nationwide registers. The annual SADP net days for 2 years before through 5 years after diagnosis date were computed. A group-based trajectory model was used to depict SADP trajectories. Associations between trajectory membership, and sociodemographic and clinical variables were tested by chi^2^ test and multinomial logistic regression.

**Results:**

Four trajectories of SADP days/year for CRC survivors were identified: “only increase around diagnosis” (52% of all), “slight increase after diagnosis” (27%), “high then decrease moderately after diagnosis” (13%), and “constantly very high” (8%). Educational level, Charlson’s Comorbidity Index, and prediagnostic mental disorders were the strongest factors determining the SADP trajectory groups. In references, three trajectories (“constantly low” (80% of all), “constantly moderate and decrease gradually” (12%), and “very high then decrease overtime” (8%)) were identified.

**Conclusion:**

Approximately 80% of CRC survivors return to a low level of SADP at 5 years postdiagnosis. Prediagnostic status of mental disorders, somatic comorbidity, and low educational level are good indicators of future high SADP levels for them. CRC survivors will benefit from early rehabilitation programs with identified risk factors.

## Introduction

Colorectal cancer (CRC) is the third most common cancer in the world and tends to affect more people in their working-age [1–3]. In Sweden, people in ages 18–65 account for approximately 30% of new CRC cases per year [4]. With early detection and improved treatment [5,6], an increasing number of CRC patients have a prolonged survival [7,8]. Consequently, for increasing number of working-aged CRC survivors, issues regarding sickness absence (SA) and disability pension (DP) are becoming of higher importance. Knowledge pertaining aspects of future work, SA, and DP in CRC survivorship will be more needed, in order to take adequate rehabilitation measures [9–13].

After treatment completion, CRC patients may still have persistent late- and long-term effects of their cancer and cancer treatment, including bowel dysfunction, peripheral neuropathy, urogenital dysfunction, and even mental disorders [14–16]. As these late effects may persist and worsen survivors’ long-term health status. The impact of these late effects on survivors’ work capacity may differ over time, as well as between survivors of different characteristics. SADP measures based on prospectively recorded register data can help quantify the length and occurrence of SADP in CRC survivors. Furthermore, the analysis of actual SADP trajectories can help identify high-risk groups that might be helped by early rehabilitation measures [17], and assist later interventions regarding SADP and return-to-work among CRC survivors. However, CRC survivors’ pre- and postdiagnosis SADP has yet been elucidated using such analysis.

By using this matched cohort study, we aimed to: 1) longitudinally illustrate different trajectories of SADP days/year in CRC survivors identified from the Swedish nationwide registers, from 2 years before to 5 years after their diagnosis; 2) evaluate factors (socio-demographic and disease-related) associated with being in different identified trajectories.

## Methods

We conducted a population-based longitudinal matched cohort study with incident CRC patients and their matched references from the population. Anonymized microdata from the following nationwide registers were obtained, linked at individual level using the ten-digit personal identity numbers assigned to all residents in Sweden [18].

### From the National Board of Health and Welfare

The Swedish Cancer Register [19] (for the identification of cancer diagnosis, diagnosis date, and cancer stage); The National Patient Register [20] (for the in-/and specialized out-patient visits 3 years prediagnosis); The Cause of Death Register [21] (for death date); The Prescribed Drug Register [22] (for purchasing prescribed psychiatric medication).

### From statistics Sweden

The longitudinal Integrated Databases for Health Insurance and Labour Market Studies [23] (LISA, for the socio-demographic information including birth year, educational level, birth country [born in Sweden or not], emigration, etc.).

### From the Swedish social insurance agency

The Microdata for Analyses of Social Insurance [24] (MiDAS), for all DP and all SA spells >14 days regarding: start/end date, grade (full- or part-time) and diagnoses.

Thereafter, we identified all 6679 people diagnosed with a first primary CRC diagnosis in Sweden in 2008–2011 when aged 18–62 years. We used International Classification of Diseases, the tenth revision (ICD-10) [25], codes C18 and C19-20 to identify colon and rectal cancer, respectively. From LISA, we then randomly selected four matched population references per patient. The prerequisites for the reference selection were that they were alive and registered in Sweden the year before the diagnosis year of the index person and did not have any previous record of CRC before the diagnosis date of the index person. A number of 26,716 references were matched to the index patient by sex, age, birth country, and educational level. After the initial identification, we then kept only those who had survived and lived in Sweden for 5 years postdiagnosis, together with their references who lived in the country during this period. Consequently, we had 4735 CRC survivors (2838 (60%) with colon cancer and 1897 with rectal cancer) and 18,230 references for the analyses.

#### Outcome measures

The outcomes of interest were SA and DP. In Sweden, SA can be granted to residents ≥16 years with an income from work or unemployment benefit, whose work capacity is reduced due to disease or injury. DP can be granted to people aged 19–64 years with long-term or permanent work incapacity due to disease or injury.

SA and DP can be granted at four levels of ordinary work hours (25%, 50%, 75%, and 100%), assessed by certified physicians based on judgement of individual’s conditions and work capacity. Net days of SA and DP were therefore calculated: e.g., two gross days of 50% SA or DP was equal to one net day.

#### Characteristics

Socio-demographic covariates included age, diagnosis/reference year, educational level, country of birth, etc. (detailed categorization in Table 1). Previous SA and DP was defined as the number of SA and DP days during the 12 months before the diagnosis date.

**Table 1:** Descriptive statistics for the study population.

Characteristics	Colon cancer survivors n (%)	Colon cancer references n (%)	Rectal cancer survivors n (%)	Rectal cancer references n (%)
	2838 (100)	10919 (100)	1897 (100)	7311 (100)
**Sex**				
*Women*	1380 (48.6)	5356 (49.1)	899 (47.4)	3495 (47.8)
*Men*	1458 (51.4)	5563 (50.9)	998 (52.6)	3816 (52.2)
**Diagnosis year**				
*2008*	734 (25.9)	2816 (25.8)	473 (24.9)	1814 (24.8)
*2009*	740 (26.1)	2837 (26.0)	460 (24.2)	1758 (24.0)
*2010*	657 (23.2)	2537 (23.2)	480 (25.3)	1864 (25.5)
*2011*	707 (24.9)	2729 (25.0)	484 (25.5)	1875 (25.6)
**Diagnosis age**				
*18–50 years*	788 (27.8)	3062 (28.0)	483 (25.5)	1877 (25.7)
*51–55 years*	533 (18.8)	2070 (19.0)	405 (21.3)	1580 (21.6)
*56–60 years*	978 (34.5)	3740 (34.3)	637 (33.6)	2448 (33.5)
*61–62 years*	539 (19.0)	2047 (18.7)	372 (19.6)	1406 (19.2)
**Birth country**				
*Sweden*	2381 (83.9)	9233 (84.6)	1586 (83.6)	6140 (84.0)
*Outside of Sweden*	457 (16.1)	1686 (15.4)	311 (16.4)	1171 (16.0)
**Educational level**				
*Elementary (≤9 years)*	594 (20.9)	2249 (20.6)	368 (19.4)	1399 (19.1)
*High school (10–12 years)*	1290 (45.5)	5002 (45.8)	894 (47.1)	3442 (47.1)
*College/university (>12 years)*	933 (32.9)	3602 (33.0)	627 (33.1)	2443 (33.4)
*Missing*	21 (0.7)	66 (0.6)	8 (0.4)	27 (0.4)
**Stage**				
*0+I*	1032 (36.4)	-	915 (48.2)	-
*II*	727 (25.6)	-	350 (18.5)	-
*III*	657 (23.2)	-	395 (20.8)	-
*IV*	121 (4.3)	-	58 (3.1)	-
*Missing*	301 (10.6)	-	179 (9.4)	-
**Charlson’s Comorbidity Index**				
*0*	2271 (80.0)	9867 (90.4)	1505 (79.3)	6601 (90.3)
*1*	286 (10.1)	649 (5.9)	149 (7.9)	446 (6.1)
*>1*	281 (9.9)	403 (3.7)	243 (12.8)	264 (3.6)
**Previous mental disorders**				
*No*	2308 (81.3)	9169 (84.0)	1577 (83.1)	6161 (84.3)
*Yes*	530 (18.7)	1750 (16.0)	320 (16.9)	1150 (15.7)
**Previous sickness absence days (in the 12 months before diagnosis date)**				
*0 days*	1955 (68.9)	9783 (89.6)	1528 (80.5)	6552 (89.6)
*>0–30 days*	553 (19.5)	405 (3.7)	198 (10.4)	270 (3.7)
*>30–90 days*	187 (6.6)	373 (3.4)	91 (4.8)	257 (3.5)
*>90–180 days*	78 (2.7)	172 (1.6)	40 (2.1)	121 (1.7)
*>180 days*	65 (2.3)	186 (1.7)	40 (2.1)	111 (1.5)
**Previous disability pension days (in the 12 months before diagnosis date)**				
*0 days*	2332 (82.2)	9253 (84.7)	1579 (83.2)	6153 (84.2)
*>0 days*	506 (17.8)	1666 (15.3)	318 (16.8)	1158 (15.8)

Stage was classified based on the information of T, N, and M from the Cancer Register, into stage 0, I-IV, and missing [26]. If T and/or N and M were missing or classified as X (assessment not possible), stage was set to missing. If more than one entry of the same type of cancer diagnosis (colon and rectal cancer, respectively) was found in the register within 30 days, the most advanced staging was used.

Further, the Charlson Comorbidity Index [27] (CCI, excluding benign and malignant tumors) was calculated based on in- and specialized out-patient visit records (from the National Patient Register) within the 3 years before the time of cancer diagnosis date for survivors and their references. Similarly, prediagnostic mental morbidity during the same period was defined as having had healthcare with ICD-10 codes of “F00-F99” or “Z73” or having bought any prescribed psychiatric medication.

#### Statistical analyses

The study population was followed from the date of diagnosis for the patients and matching date for the references, respectively; until the date of reaching old-age pension (turning 65 years), or December 31, 2016, whichever came first. Descriptive statistics of the study population were presented by colon and rectal cancer.

To identify subgroups of individuals with distinct trajectories of SADP before and after CRC diagnosis, we applied a group-based trajectory model [28]. This model was used to measure trajectories of SADP days/year in both CRC survivors and their matched references, in the 2 years before diagnosis date (Y_-2_ and Y_-1_) to 5 years after diagnosis date (Y_+1_—Y_+5_, accordingly). The Bayesian information criterion was used to determine the model of best fit. We applied this model to distinguish patterns of postdiagnostic SADP in colon and rectal cancer separately, and in diagnosis age groups of ≤57 and >57 years, respectively.

The distribution of socio-demographics and clinical characteristics in each SADP trajectory group among the CRC survivors were calculated and tested using Pearson’s ꭓ^2^ test and multinomial logistic regression. Additionally, a likelihood ratio test was performed to assess the associations between the abovementioned variables and type of trajectory group in the full model. Then, the Nagelkerke pseudo R^2^ was applied to estimate the strength of these associations. To achieve that, each variable was consecutively included and then excluded from the full model to calculate the R^2^ differences. The values of R^2^ indicated the contribution of a given variable to the full model.

All statistical analyses were conducted using SAS 9.4 (SAS Institute, Cary, NC).

The project was approved by the Regional Ethical Review Board of Stockholm, Sweden.

## Results

Descriptive statistics of socio-demographic and clinical characteristics are presented by colon and rectal cancer, respectively, along with their references (Table 1).

**Fig 1:**
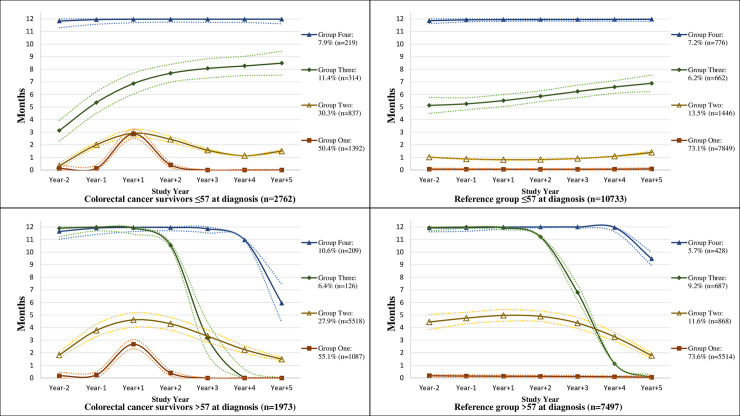
Trajectories of the mean number of net months on sickness absence and/or disability pension per year with 95% confidence intervals; from 2 years prior (Year_-1_ and Year_-2_) to 5 years after colorectal cancer diagnosis date and matching date for colorectal cancer survivors and their references, respectively (Year_+1_, Year_+2_, Year_+3,_ Year_+4,_ and Year_+5_). Dotted lines indicate confidence intervals.

Four trajectories of SA/DP days were identified for the study period among the CRC survivors, and three trajectories among their references (Fig 1). The majority of CRC survivors (52% of all) were identified in the trajectory group here called “Only increase around diagnosis”. They had almost 0 SADP days/year in Y_-2_ and Y_-1_, even though they had a sudden spike of SADP level of 90 days/year during Y_+1_, the amount of SADP returned to the same level as before diagnosis from Y_+2_. Approximately another one fourth of CRC survivors belonged to the trajectory group of “slight increase after diagnosis”. Survivors in this group had elevated SADP around 60 days already during Y_-1_. During Y_+1_, their SADP slightly increased to 90 days/year, which gradually returned to around 50 days/year during Y_+2_ and Y_+5_. In the trajectory of “high then gradually decrease”, 605 survivors (13% of all survivors) were seen having a level of around 200 SADP days/year already in Y_-2_, which then increased to 250 days/year in Y_+1_. The level stabilized before it gradually dropped to around 100 days/year in Y_+5_. In the group of “constantly very high”, (7.5% of all survivors) had a constant high number of SADP days for all seven years. As for the references, over 80% had almost no SADP days from Y_-2_ to Y_+5_. A proportion of 12% had high level of SADP (around 200 days/year), which dropped slowly over time. A similar proportion as in the survivor group (8% of all) were constantly on SADP all of the first six years, which then decreased towards the last year of follow-up.

Stratifying by colon and rectal cancer, we observed a similar trend as in the group of all CRC survivors and their references, although the number of survivors were smaller and only three trajectories were identified for survivors of colon and rectal cancer, respectively (S1 Fig).

Among CRC survivors, the distribution of characteristics in each trajectory group, and the associations between each variable and the trajectory group membership were shown (Table 2). Individuals in the trajectory group “Only increase around diagnosis” tended to be men, born in Sweden, with higher education, having none previous somatic/psychiatric comorbidity and having none/or low previous SADP days. The group “Slightly increase after diagnosis” and “High then decrease moderately” more likely consisted of survivors of having more previous somatic/psychiatric comorbidity and higher levels of prediagnostic SADP days. In the group “Constantly very high”, the majority of individuals were born outside of Sweden, had low educational level, diagnosed at a later age, having larger number of prediagnostic somatic/psychiatric comorbidity and having higher level of prediagnostic SADP days. In the unadjusted analyses demonstrated by the results from Pearson’s ꭓ^2^ test and Log-likelihood test, all variables except for diagnosis year and type of cancer were significantly associated with the trajectory membership. However, the associations changed after mutual adjustment in the full model. The Nagelkerke pseudo R^2^ for the full model was 0.1877. The differences of R^2^ between the full model and the individual models without the variable of interest were the largest for prediagnostic mental disorders (R^2^ = 0.067), Charlson’s Comorbidity Index (R^2^ = 0.029) and educational level (R^2^ = 0.022). The large values observed in the three variables indicated as having important roles in their trajectory group membership determination, while the most important determinant being having mental disorders prediagnosis.

**Table 2:** Distributions and associations of different characteristics in each trajectory group in colorectal survivors (n = 4735).

Characteristics	Only increase around diagnosis n (%)	Slight increase after diagnosis n (%)	High then moderate increase gradually n (%)	Constantly very high n (%)	Pearson's ꭓ^2^ (*p*-value)	Log-likelihood test ꭓ^2^ (*p*-value)	Diff in R^2^
**Total**	**2481 (100)**	**1194 (100)**	**605 (100)**	**355 (100)**			
**Sex**							
*Women*	1073 (43.2)	671 (51.9)	340 (56.2)	195 (54.9)	53.2 (<0.001)	53.3 (<0.001)	0.009
*Men*	1408 (56.8)	623 (48.1)	265 (43.8)	160 (45.1)			
**Diagnosis year**							
*2008*	625 (25.2)	328 (25.3)	167 (27.6)	87 (24.5)	12.2 (0.20)	12.2 (0.21)	0.007
*2009*	616 (24.8)	310 (24.0)	166 (27.4)	108 (30.4)			
*2010*	606 (24.4)	318 (24.6)	130 (21.5)	83 (23.4)			
*2011*	634 (25.6)	338 (26.1)	142 (23.5)	77 (21.7)			
**Age at diagnosis**							
*≤57 years*	1414 (57.0)	869 (67.2)	261 (43.1)	218 (61.4)	102.1 (<0.001)	102.2 (<0.001)	0.015
*>57 years*	1067 (43.0)	425 (32.8)	344 (56.9)	137 (38.6)			
**Birth country**							
*Sweden*	2154 (86.8)	1073 (82.9)	487 (80.5)	253 (71.3)	63.3 (<0.001)	57.9 (<0.001)	0.008
*Outside of Sweden*	327 (13.2)	221 (17.1)	118 (19.5)	102 (28.7)			
**Educational level**							
*≤9 years + missing*	447 (18.0)	246 (19.0)	174 (28.8)	124 (34.9)	144.7 (<0.001)	148.3 (<0.001)	0.022
*10–12 years*	1090 (43.9)	619 (47.8)	298 (49.3)	177 (49.9)			
*>12 years*	944 (38.0)	429 (33.2)	133 (22.0)	54 (15.2)			
**Type of cancer**							
*Colon*	1494 (60.2)	766 (59.2)	355 (58.7)	223 (62.8)	2.0 (0.57)	2.0 (0.57)	0.007
*Rectal*	987 (39.8)	528 (40.8)	250 (41.3)	132 (37.2)			
**Stage**							
*0+I*	1060 (42.7)	494 (38.2)	232 (38.3)	161 (45.4)	57.2 (<0.001)	53.9 (<0.001)	0.009
*II*	569 (22.9)	304 (23.5)	138 (22.8)	66 (18.6)			
*III*	555 (22.4)	293 (22.6)	130 (21.5)	74 (20.8)			
*IV*	55 (2.2)	61 (4.7)	47 (7.8)	16 (4.5)			
*Missing*	242 (9.8)	142 (11.0)	58 (9.6)	38 (10.7)			
**Charlson’s Comorbidity Index (in three years prediagnosis)**							
*0*	2097 (84.5)	1062 (82.1)	388 (64.1)	229 (64.5)	233.9 (<0.001)	199.0 (<0.001)	0.029
*1*	162 (6.5)	104 (8.0)	92 (15.2)	77 (21.7)			
*>1*	222 (8.9)	128 (9.9)	125 (20.7)	49 (13.8)			
**Mental disorders (in three years prediagnosis)**							
*No*	2252 (90.8)	1071 (82.8)	385 (63.6)	177 (49.9)	517.6 (<0.001)	455.0 (<0.001)	0.066
*Yes*	229 (9.2)	223 (17.2)	220 (36.4)	178 (50.1)			

**Fig 2:**
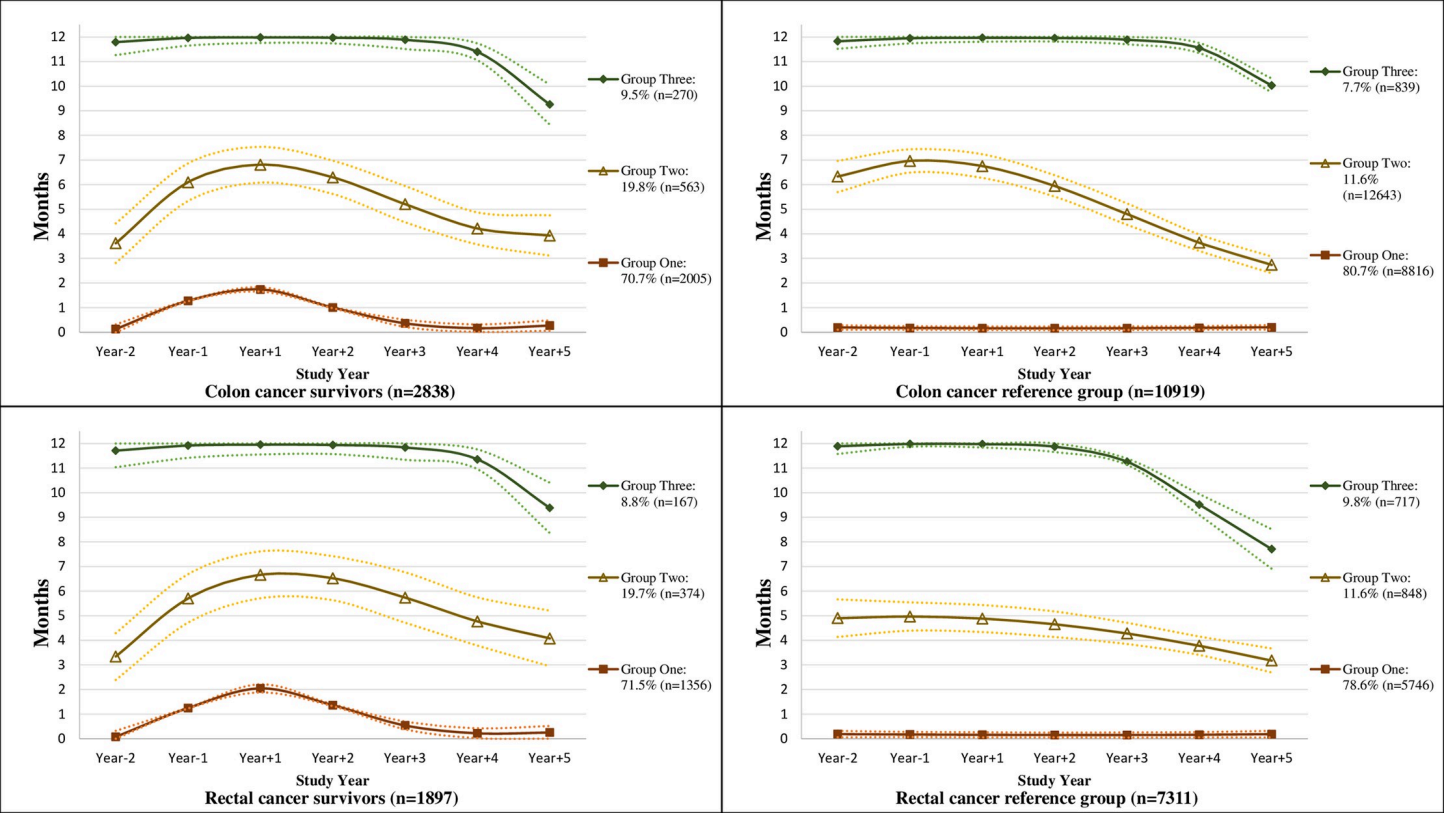
Age-specific (≤57 and >57 years of age) trajectories of the mean number of net months on sickness absence and/or disability pension per year with 95% confidence intervals; from 2 years prior (Year_-1_ and Year_-2_) to 5 years after colorectal cancer diagnosis date and matching date for colorectal cancer survivors and their references, respectively (Year_+1_, Year_+2_, Year_+3,_ Year_+4,_ and Year_+5_). Dotted lines indicate confidence intervals.

After stratification by age, the trajectory patterns varied moderately between CRC survivors younger and older than 57 of age (Fig 2). Similar to the overall survivors, around 50% of people in each age subgroup belonged to the trajectory of “only increase around diagnosis (indicated as “Group 1”)” with almost 90 SADP days as the peak value during Y_+1_. Likewise, about 30% were included in the group of “slightly increase after diagnosis (indicated as “Group 2”)”, however, the general number of SADP days in those >57 years was higher (with its peak value of 150 days in Y_+1_) than in the group ≤57.

## Discussion

The large-scale population-based longitudinal study illustrated the dynamic trajectories of SADP levels in first-diagnosed CRC survivors and their general population references over a timespan from Y_-2_ to Y_+5_. Among the CRC patients who survived in Sweden five years postdiagnosis, over half had a peak of 90 SADP days in Y_+1_ which then decreased to 0 in Y_+5_. Another 30% of the CRC survivors with prediagnosis moderate SADP would have a moderate increase right after diagnosis which then returned to around 60 days/year in Y_+5_. Lower educational level, having prediagnostic somatic comorbidity and mental disorders were all factors that were associated with how survivors’ SADP trajectories changed over time.

From the trajectory analyses, the majority of CRC survivors (80%) in our study had slightly higher numbers of SADP days compared to the general population over a 5-year span. The overall finding was in line with previous studies based on CRC survivors from the Netherlands [11] and rectal cancer survivors from Sweden [29]. Previous studies on work situations on CRC survivors were often limited in being only questionnaire-based [30–33] having small sample size [30–35], focusing not colon and rectal cancer together [29,31,36], with short follow-up (less than 2 years) [13,30–33,35], not quantifying its outcomes (with only binary outcome of return-to-work or not) [13,30–34,37], without information of DP [13,30–33,35,37]. For the first time, we also showed that among all CRC survivors, half of them only had an increase of SADP days of 90 days during Y_+1_, which then returned to almost none, just as before the diagnosis. The increase of SADP days among another 30% of survivors remained at a rather low level of 60 days/year in Y_+5_. Our study was based on survivors diagnosed in 2008–2011 who received recent modern treatment strategies. Existing studies on SADP in CRC survivors are mostly based on patients diagnosed earlier [34], or only on rectal cancer survivors [29,36]. Further, as for the length of absent days from work, one study on middle-aged CRC survivors (45–64 of age at diagnosis) from Australia showed that 1/3 of the sampled survivors stopped working and the majority of survivors who returned to work took approximately 90 days off work during Y_+1_ [33], which was also consistent with our findings.

Previous studies found that advanced cancer stage [29,34], extensive surgical operation [29,36,38], postoperative complications [29,36,38], previous SA/DP [29,36], previous unemployment [38], and lower educational level [34,36], were associated with a higher risk of SADP in CRC survivors at different time points (ranging from 6 months up to 10 years postdiagnosis). Presently, we found that educational level, having prediagnostic somatic, and having mental morbidity were strongly associated with being in SADP trajectories with higher numbers of SADP overtime, while having prediagnostic mental morbidity being the most prominent one. We further highlighted the importance of comorbidity, particularly mental comorbidity, rather than of cancer stage. This finding is in line with previous studies that show somatic and mental morbidities play significant roles during CRC survivorship [14–16], which is highly dependent of one’s previous morbidity. Another underlying explanation to the limited influence of cancer stage on SADP trajectory membership may be that the survivors included all survived 5 years after diagnosis/treatment, which is rather a standard time to classify that one is totally free of CRC. Hence, for long-term CRC survivors, the factors that impact his/her work situation may rather not be related to the initial CRC and its related treatment, but their previous or later morbidity.

### Strengths and limitations

One of our strengths is the use of longitudinal, nationwide, high-quality population-based register data provided by different authorities [20,23]. These resources have also enabled us to have a matched reference group from the general population and comprehensive information of different variables and study outcomes over a long follow-up. Thus, we included all CRC survivors fulfilling the inclusion criteria from the entire country (instead of a sample), allowing sub-group analyses, with no losses to follow-up. All the data were administrative, rather than self-reports sometimes hampered by recall bias. Other strengths are that we could follow the individuals from actual inclusion date and included also DP days. This is the first study to illustrate SADP levels using trajectory method in CRC survivors pre- and postdiagnosis, facilitating the understanding of trends and variations of SADP over time in survivors overall, and by characteristics. Nevertheless, one limitation was the information about treatment was unavailable, hampering further study the treatment-induced long-term SA/DP. Future studies may benefit from inclusion of these additional variables, being able to investigate the impact of specific treatment on CRC survivors’ SA/DP. Additionally, we lacked information of SA spells ≤14 days, which could have led to an underestimation of the amount of SADP days. However, the situation also holds among the references and it is the long-term spells that stand for the absolute majority of SA days. Moreover, we chose to use the Charlson Comorbidity Index, although it was originally designed to measure the impact of comorbidity burden on mortality post-hospitalization. This was because our main interest was not to study whether the comorbidity was clinically relevant to CRC survivors, but to SA and DP. As the Index is well known and, therefore, possible to use by other researchers, we applied it instead of other measures of comorbidity- or multi-morbidity, which might be a more appropriate term for what we measured. We had no information on where in Sweden those included lived, however, great effort is taken to provide equal access and quality in cancer care in different regions in Sweden as well as regarding equal assessments of right to SA and DP benefits.

Conclusively, approximately 80% of CRC survivors experienced a low level of SADP days prediagnosis. Although they experienced a peak of having 90 days of SADP during Y_+1_, they gradually returned to a rather low level of having 0–60 days/year in Y_+5_. Our results also highlighted the heterogeneity of SADP days in CRS survivors and the importance of prediagnostic somatic and mental comorbidity, and educational level in determining 5-year CRC survivors’ work situation, rather than the initial cancer stage itself.

## Supporting information

S1 Fig Specific trajectories of the mean number of net months on sickness absence and/or disability pension per year with 95% confidence intervals; from 2 years prior (Year_-1_ and Year_-2_) to 5 years after colon/rectal cancer diagnosis date and matching date for colon/rectal cancer survivors and their references, respectively (Year_+1_, Year_+2_, Year_+3,_ Year_+4,_ and Year_+5_). Dotted lines indicate confidence intervals.
(TIF)
