# Supplementary figures and images for: Trajectories of sickness absence and disability pension before and after colorectal cancer: A Swedish longitudinal population-based matched cohort study
Source: PLoS One. 2021 Jan 7;16(1):e0245246. doi: 10.1371/journal.pone.0245246 (PMC7790369; doi:10.1371/journal.pone.0245246)

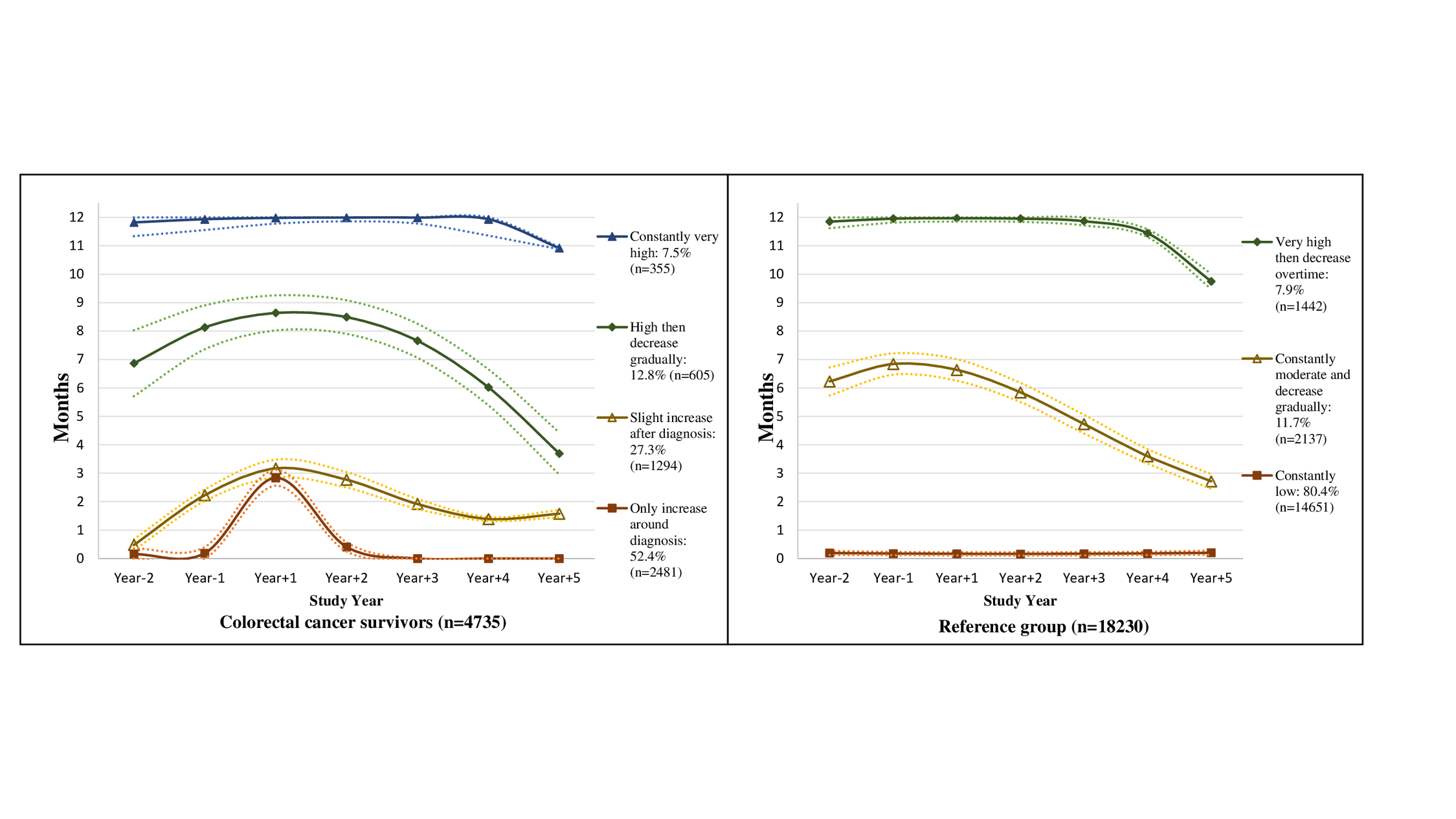

Supplement: S1 Fig — Dotted lines indicate confidence intervals. (TIF) [file pone.0245246.s001.tif]
